# Development and validation of a cardiometabolic multimorbidity prediction model in middle-aged and older adults

**DOI:** 10.1038/s41598-026-44213-0

**Published:** 2026-03-12

**Authors:** Hongjiang Li, Xin Ma, Tingting Cui, Wenhui He, Liping Zhu, Hongling Zhang

**Affiliations:** 1 Present Address: Department of General Practice, First Clinical Medical College, Shanxi Medical University, Taiyuan, Shanxi China; 2 Present Address: Department of General Practice, First Hospital of Shanxi Medical University, Taiyuan, Shanxi China

**Keywords:** Cardiometabolic multimorbidity, Risk prediction, CHARLS, Cohort study, Diseases, Health care, Medical research, Risk factors

## Abstract

Cardiometabolic multimorbidity (CMM) is a prevalent syndrome among middle-aged and older adults, significantly impairing quality of life and imposing substantial health and economic burdens on China’s aging healthcare system. The development of timely predictive models is crucial for enabling early intervention. This study aimed to integrate multidimensional data from the China Health and Retirement Longitudinal Study (CHARLS) to develop an effective predictive model for assessing the five-year risk of CMM onset, thereby facilitating early intervention and management for individuals at risk in China. We analyzed data from the 2015 to 2020 CHARLS surveys, involving 5,388 middle-aged and older adults initially free of CMM. The dataset was randomly split into a training set (70%) and a validation set (30%). Key predictors were identified from 31 potential variables using LASSO regression with 10-fold cross-validation. Selected predictors underwent correlation analysis and were used to construct both Extreme Gradient Boosting (XGBoost) and Logistic Regression (LR) models. Variable contributions in the XGBoost model were interpreted using SHapley Additive exPlanations (SHAP) values, while the LR model was visualized via a nomogram. The performance of the superior model was evaluated using receiver operating characteristic curves, calibration curves, and decision curve analysis. LASSO regression screened significant variables from the initial 31 candidates. Correlation analysis ultimately identified nine key predictors: systolic blood pressure, BMI, fasting blood glucose, total cholesterol, triglycerides, uric acid, age, comorbidities, and pain. The logistic regression model demonstrated superior and more stable predictive performance on the validation set, with an area under the curve (AUC) of 0.732 (95% CI: 0.703–0.761). Calibration curves indicated reliable predictive accuracy, and decision curve analysis established the clinical net benefit across a range of risk thresholds. This study developed and validated a reliable, clinically applicable logistic regression model, supplemented by a nomogram, for assessing the five-year risk of CMM in Chinese middle-aged and older adults. The model effectively identifies high-risk individuals, supporting targeted early intervention and management strategies to alleviate the health and economic burdens associated with CMM in this population.

**Supplementary Information:**

The online version contains supplementary material available at 10.1038/s41598-026-44213-0.

## Introduction

The accelerating pace of global population aging, coupled with shifts in lifestyle and dietary habits, has contributed to a rising prevalence of cardiovascular metabolic diseases (CMDs), presenting a significant public health challenge worldwide^1,2^. Notably, these conditions frequently co-occur. Large-scale prospective cohort studies have established a high prevalence of comorbidity between cardiovascular disease and glucose-lipid metabolism abnormalities, with a growing number of individuals affected by two or more CMDs^3–7^. The situation is particularly severe in China, where approximately 500,000 annual cardiovascular deaths are attributable to abnormal glucose metabolism^8^. This convergence has established the co-occurrence of cardiovascular and metabolic diseases as a major public health issue, a condition termed cardiometabolic multimorbidity (CMM)^9^. CMM is defined as the simultaneous presence of two or more CMDs^10^. Compared to a single CMD, CMM exerts a greater adverse impact on health outcomes, with affected patients experiencing a reduction in life expectancy approximately twice that of those with a single condition^11^. CMM also significantly diminishes quality of life^12^, substantially increases healthcare expenditure^13^, and elevates the risk of neurological disorders such as cognitive impairment^14^. These stark statistics underscore the insufficiency of previous research focusing on individual diseases (e.g., hypertension, diabetes, stroke, heart disease, dyslipidaemia) to address the current threat posed by the high prevalence of CMM to the health of middle-aged and older adults in China. Consequently, there is an urgent need for tools capable of early CMM risk identification to enable proactive intervention and management for high-risk populations, thereby mitigating the associated health and economic burdens.

Although CMM prediction is crucial, existing research has largely been confined to examining its association with single or composite indicators^15–17^, such as the triglyceride-glucose index, BMI, waist circumference, or waist-to-height ratio. This approach fails to account for the complex pathophysiology of CMM development and is insufficient for achieving precise, individualized prediction. More significantly, there is a current lack of personalized risk assessment tools that can effectively integrate multidimensional information for direct use by healthcare professionals in primary care and clinical practice. This gap hinders the early screening and management of populations at high risk for CMM.

Within the methodological domain of predictive modeling, machine learning algorithms like XGBoost have demonstrated formidable predictive capabilities across numerous medical forecasting tasks^18,19^ due to their capacity to handle complex, non-linear relationships. However, their “black-box” nature constrains clinical application; despite techniques like SHapley Additive exPlanations (SHAP) that enhance interpretability, model complexity remains a barrier to widespread clinical adoption. In this context, nomogram visualizations based on logistic regression models offer a practical and interpretable alternative.

To address these research gaps, this study aimed to develop and validate prediction models for the five-year risk of CMM onset among Chinese middle-aged and older adults. Utilizing longitudinal data from the nationally representative China Health and Retirement Longitudinal Study (CHARLS) between 2015 and 2020, we employed both the Extreme Gradient Boosting (XGBoost) algorithm and the Logistic Regression (LR) algorithm. Our objective was to provide healthcare professionals with a convenient and reliable tool for the precise, individualized assessment of CMM risk, thereby informing clinical decision-making and offering practical insights for the development of clinical guidelines and health policies.

## Methods

### Study population

Data for this study were obtained from the China Health and Retirement Longitudinal Study (CHARLS), a national survey initiated by the National School of Development at Peking University in 2011. CHARLS employed a stratified, multistage probability proportional to size sampling method, covering 150 counties and 450 villages/urban communities across 28 provinces, ultimately involving 10,257 households and 17,708 individuals. Follow-up surveys were conducted in 2013, 2015, 2018, and 2020. Detailed study information is available at http://charls.pku.edu.cn/, and the data are publicly accessible.

This study utilized data from the 2015 wave of the China Health and Retirement Longitudinal Study (CHARLS) as the baseline. This wave was selected for three primary reasons: (1) it provided the most comprehensive and reliable contemporaneous clinical and laboratory measurements required for model development; (2) its temporal proximity enhances the model’s applicability to contemporary and future middle-aged and older populations in China; and (3) it allowed for a sufficient 5‑year follow‑up period (through 2020) to ascertain incident cases of cardiometabolic multimorbidity. From an initial pool of 21,097 participants in the 2015 survey, the final analytical cohort comprised 5,388 individuals after applying the following exclusion criteria: (1) absence of blood test-related information; (2) age below 45 years (middle-aged and older adults were defined as ≥ 45 years, consistent with classifications from the World Health Organization and the Chinese government); (3) a diagnosis of CMM in 2015 or lack of CMM-related information at baseline; (4) missing CMM-related data during the 2018 and 2020 follow-up surveys; and (5) other relevant data omissions.

### Sample size considerations

The sample size for this study was determined by the availability of participants meeting the inclusion criteria in the CHARLS 2015 wave and was evaluated according to the events‑per‑variable (EPV) principle^20^. The final analytical cohort comprised 5,388 individuals, with a training set of 3,771 participants. Based on the EPV guideline, a training set containing at least 90 events is recommended to support the nine predictor variables included in the model. Given the observed event incidence of 20.12%, the minimum required sample size for the training set would be 448 individuals.

### Ethical considerations

The study was approved by the Biomedical Ethics Review Committee of Peking University (IRB No. 00001052–11014). All participants provided written informed consent. CHARLS adheres to the ethical principles of the Declaration of Helsinki and China’s Personal Information Protection Law. The CHARLS database implements stringent privacy protection and anonymization protocols during data collection and processing to ensure the security of participants’ personal information.

### Determination of cardiovascular metabolic multi-morbidity (CMM)

Cardiovascular metabolic diseases (CMDs) in this study included hypertension, diabetes, dyslipidaemia, stroke, and heart disease. CMM was defined as the presence of two or more of these CMDs^10^.

Hypertension was defined as: mean systolic blood pressure (SBP) ≥ 140 mmHg, mean diastolic blood pressure (DBP) ≥ 90 mmHg, a prior physician diagnosis of hypertension, or current use of antihypertensive medication.

Dyslipidaemia was defined as: total cholesterol (TC) ≥ 240 mg/dL, low-density lipoprotein cholesterol (LDL-C) ≥ 160 mg/dL, high-density lipoprotein cholesterol (HDL-C) < 40 mg/dL, triglycerides (TG) ≥ 200 mg/dL, or a prior physician diagnosis of dyslipidaemia.

Diabetes was defined as: fasting plasma glucose (FPG) ≥ 7.0 mmol/L, glycated haemoglobin (HbA1c) ≥ 6.5%, current use of antidiabetic medication, or a self-reported physician diagnosis of diabetes or hyperglycaemia.

Identification of heart disease or stroke primarily relied on self-reported data from baseline and follow-up surveys, or recorded use of cardiovascular medications. Standardized questions were used: “Have you ever been diagnosed by a doctor with coronary heart disease, angina pectoris, congestive heart failure, or any other heart condition?” and “Have you been diagnosed by a doctor with a stroke?”

## Potential predictor variables

### Laboratory indicators

Laboratory assessments included a panel of biochemical indicators: white blood cell count, haemoglobin, fasting glucose, uric acid, creatinine, total cholesterol, triglycerides, high-density lipoprotein cholesterol, low-density lipoprotein cholesterol, C-reactive protein, and glycated haemoglobin.

### Physical examination

Physical examination comprised measurements of systolic and diastolic blood pressure, pulse rate, and respiratory function. Trained staff conducted standardized blood pressure measurements (after at least 5 min of rest in a quiet environment, using a sphygmomanometer for three readings). Respiratory function was assessed using a peak flow meter; the highest value from three attempts was recorded. Grip strength was measured for each hand using a WL-1000 dynamometer (Nantong Yuejian Physical Measurement Instrument Co., Ltd.). Two measurements were taken per hand, and the average of the highest values from both hands represented overall grip strength. Disability status was assessed using five questions regarding the presence of any disability, brain injury/intellectual disability, vision problems, hearing problems, or speech difficulties. An affirmative answer to any question classified the participant as having a disability.

### Other potential predictors

Additional predictor variables, identified from prior literature as potential correlates of CMD^21–26^, included: age, gender, ethnicity, retirement status, marital status, residential area, educational attainment, daily sleep duration, alcohol and tobacco consumption, BMI, waist circumference, pain, and comorbidities (excluding individuals with established CMM at baseline). Pain was assessed based on the presence of pain in any of the following regions: headache, neck, arm, shoulder, wrist, finger, chest, stomach, back, lumbar, hip, leg, knee, ankle, or toe. Comorbidities encompassed a history of hypertension, dyslipidaemia, diabetes or hyperglycaemia, cancer, chronic pulmonary disease, liver disease, heart disease, stroke, kidney disease, digestive system disorders, emotional and mental health issues, memory-related conditions, arthritis or rheumatic diseases, and asthma. BMI was calculated from measured weight and height. The comorbidity survey was based on the standard CHARLS questionnaire design.

### Statistical analysis

This study aimed to develop a tool for estimating the absolute 5‑year risk of CMM onset among individuals free of CMM at baseline. Accordingly, we employed both logistic regression and XGBoost to model the binary outcome (CMM within 5 years), rather than time‑to‑event approaches (e.g., Cox regression or time‑dependent XGBoost). Both methods provide probability estimates for a fixed time horizon, which are readily interpretable in clinical practice. All predictors were defined at baseline (2015), aligning with the design of widely implemented risk‑prediction tools such as the Framingham Risk Score, which also uses a single assessment to estimate long‑term risk (e.g., 10‑year)^27,28^.

The entire dataset was randomly partitioned into a training set (70%) and a validation set (30%). Continuous variables were described using mean and standard deviation or median and interquartile range (IQR), and compared using t-tests or Mann-Whitney U tests. Categorical variables were expressed as frequencies and percentages, and compared using χ² tests.

Variable selection was performed on the training set using LASSO regression with 10-fold cross-validation. LASSO was chosen for its ability to perform variable selection and regularization simultaneously, which helps obtain a parsimonious set of predictors during the feature screening phase, thereby reducing the risk of overfitting at this stage. Correlation analysis was then conducted to identify potential multicollinearity. Variables exhibiting high correlation were excluded to ensure model stability and parsimony, taking clinical relevance into account. The finalized variables were used to construct both XGBoost and logistic regression models. For the XGBoost model, 10-fold cross-validation was employed within a Bayesian optimization framework to identify the optimal hyperparameter combination. SHapley Additive exPlanations (SHAP) values were used to interpret variable importance and feature contributions toward CMM prediction. Four key SHAP plots were generated: (1) SHAP Bar Plot, (2) SHAP Summary Plot, (3) SHAP Dependence Plot, and (4) SHAP Force Plot. Using the variables selected by LASSO, a standard multivariable logistic regression model was fitted to obtain regression coefficients and odds ratios for each feature, which can be directly interpreted in clinical practice. The model was also visualized using a nomogram. This two‑step approach—using LASSO for objective variable selection followed by standard logistic regression for unbiased parameter estimation—ensures both model parsimony and the clinical interpretability required for a practical risk tool. The performance of both models on the validation set was compared using metrics including the area under the curve (AUC), sensitivity, specificity, accuracy, precision, and F1 score. The superior model was further evaluated using a receiver operating characteristic (ROC) curve, while calibration curves assessed the agreement between predicted probabilities and observed outcomes. Decision curve analysis (DCA) was used to evaluate the net benefit of using the prediction model across a range of clinically reasonable probability thresholds, compared with the strategies of intervening for all patients or for none.

All statistical analyses were conducted using R version 4.5.1 and SPSS version 27.0. A bilateral p-value < 0.05 was considered statistically significant.

## Results

### Baseline characteristics

**Fig. 1:**
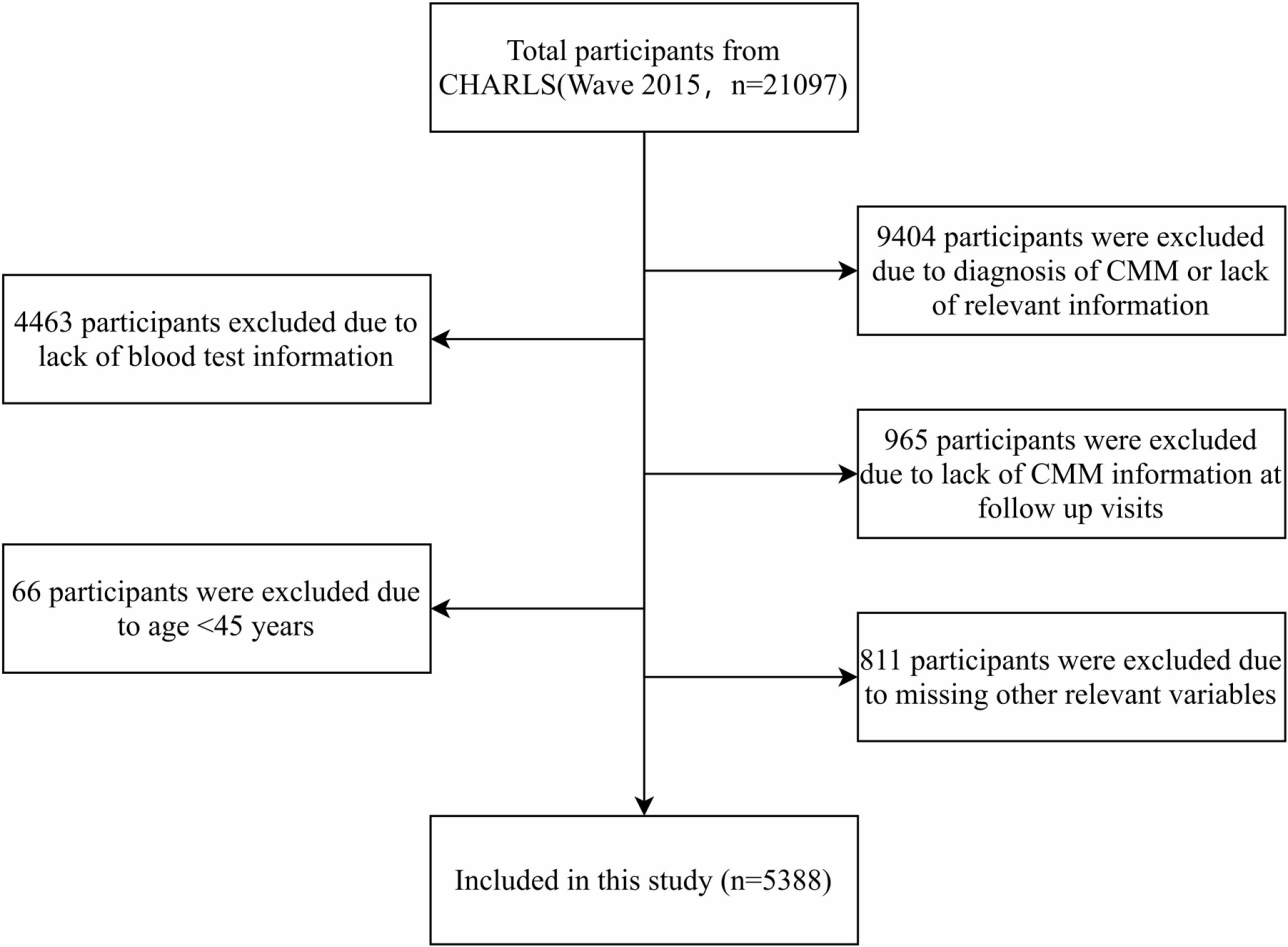
Flowchart of study participant selection.

CHARLS: China Health and Retirement Longitudinal Study; CMM: Cardiovascular Metabolic Multimorbidity.

This study analyzed 5,388 middle-aged and older adults from the 2015 CHARLS survey. Over the five-year follow-up period, 1,084 participants developed CMM, corresponding to an incidence rate of 20.12%. Among these incident cases, the most frequent disease combination was hypertension with dyslipidaemia (*n* = 221, representing 20.4% of all CMM cases). A complete breakdown of all disease combinations is provided in Supplementary Material S1. The dataset was split into a training set (*n* = 3,771; 772 CMM events) and a validation set (*n* = 1,617; 312 CMM events). Table 1; Fig. 1 detail the baseline characteristics and participant selection flow for both cohorts. Most characteristics, including key predictor variables, were well-balanced between the training and validation sets (*P* > 0.05), with the exception of gender, marital status, smoking status, respiratory function, haemoglobin, and grip strength.

**Table 1:** Baseline characteristics of the training and validation cohorts.

Variable	Overall (*n* = 5388)	Training set (*n* = 3771)	Validation set (*n* = 1617)	*P* value
Gender				0.002
Female	2833 (52.58)	2036 (53.99)	797 (49.29)	
Male	2555 (47.42)	1735 (46.01)	820 (50.71)	
Living area				0.773
Urban	1834 (34.04)	1279 (33.92)	555 (34.32)	
Rural	3554 (65.96)	2492 (66.08)	1062 (65.68)	
Ethnicity				0.333
Other ethnic groups	292 (5.42)	197 (5.22)	95 (5.88)	
Han	5096 (94.58)	3574 (94.78)	1522 (94.12)	
Marital status				0.020
Unmarried	576 (10.69)	379 (10.05)	197 (12.18)	
Married	4812 (89.31)	3392 (89.95)	1420 (87.82)	
Drinking				0.133
No	2873 (53.32)	2036 (53.99)	837 (51.76)	
Yes	2515 (46.68)	1735 (46.01)	780 (48.24)	
Smoking				0.047
No	3066 (56.90)	2179 (57.78)	887 (54.85)	
Yes	2322 (43.10)	1592 (42.22)	730 (45.15)	
Retire				0.843
No	4825 (89.55)	3379 (89.60)	1446 (89.42)	
Yes	563 (10.45)	392 (10.40)	171 (10.58)	
SBP (mmHg)	123.59 ± 18.44	123.38 ± 18.49	124.07 ± 18.32	0.212
DBP (mmHg)	73.67 ± 11.34	73.53 ± 11.50	73.98 ± 10.97	0.177
Pulse	73.41 ± 10.27	73.38 ± 10.33	73.49 ± 10.13	0.720
WC (cm)	84.85 ± 9.84	85.00 ± 9.86	84.52 ± 9.80	0.102
BMI (kg/m²)	23.43 ± 3.38	23.45 ± 3.39	23.38 ± 3.34	0.506
Respiratory function	320.00 (240.00, 400.00)	310.00 (240.00, 397.50)	320.00 (250.00, 405.00)	0.008
Sleeping (h)	6.43 ± 1.87	6.43 ± 1.87	6.42 ± 1.86	0.841
Disability				0.144
No	3882 (72.05)	2739 (72.63)	1143 (70.69)	
Yes	1506 (27.95)	1032 (27.37)	474 (29.31)	
Age (years)	60.04 ± 8.65	60.09 ± 8.70	59.93 ± 8.53	0.544
Education level				0.732
Below primary school	2278 (42.28)	1598 (42.38)	680 (42.05)	
Primary school graduate	1247 (23.14)	875 (23.20)	372 (23.01)	
Middle school graduate	1271 (23.59)	887 (23.52)	384 (23.75)	
High school graduate and above	592 (10.99)	411 (10.90)	181 (11.19)	
Comorbidities				0.681
No	1532 (28.43)	1066 (28.27)	466 (28.82)	
Yes	3856 (71.57)	2705 (71.73)	1151 (71.18)	
Pain				0.415
No	3975 (73.78)	2770 (73.46)	1205 (74.52)	
Yes	1413 (26.22)	1001 (26.54)	412 (25.48)	
Hand grip	30.39 ± 9.16	30.22 ± 9.17	30.78 ± 9.11	0.039
CMM				0.323
No	4304 (79.88)	2999 (79.53)	1305 (80.71)	
Yes	1084 (20.12)	772 (20.47)	312 (19.29)	
Blood test results				
WBC (×10⁹/L)	5.86 ± 1.76	5.89 ± 1.76	5.80 ± 1.75	0.110
GLU (mg/L)	97.34 ± 22.64	97.43 ± 23.61	97.13 ± 20.19	0.647
CREA (mg/dL)	0.80 ± 0.23	0.80 ± 0.23	0.80 ± 0.23	0.350
CHO (mg/dL)	182.87 ± 34.82	182.86 ± 34.90	182.90 ± 34.63	0.969
TG (mg/dL)	107.08 (79.65, 159.29)	107.96 (79.65, 160.18)	106.19 (79.65, 155.75)	0.356
HDL (mg/dL)	52.10 ± 11.54	52.05 ± 11.51	52.23 ± 11.63	0.599
LDL (mg/dL)	101.94 ± 28.15	101.83 ± 28.10	102.21 ± 28.27	0.655
CRP (mg/L)	1.30 (0.70, 2.30)	1.20 (0.70, 2.30)	1.30 (0.70, 2.30)	0.533
HbA1c (%)	5.79 ± 0.68	5.79 ± 0.70	5.78 ± 0.64	0.510
UA (mg/dL)	4.85 ± 1.35	4.84 ± 1.34	4.87 ± 1.38	0.451
HGB (g/dL)	13.68 ± 1.90	13.63 ± 1.87	13.80 ± 1.96	0.002

SBP: Systolic blood pressure. DBP: Diastolic blood pressure. WBC: White blood cells. HGB: Haemoglobin. TG: Triglycerides. CHO: Total cholesterol. GLU: Glucose. UA: Uric acid. CRP: C-reactive protein. HbA1c: Glycated haemoglobin. CREA: Creatinine. HDL: High-density lipoprotein cholesterol. LDL: Low-density lipoprotein cholesterol. CMM: Cardiovascular metabolic comorbidity.

### Selection of predictor variables

**Fig. 2:**
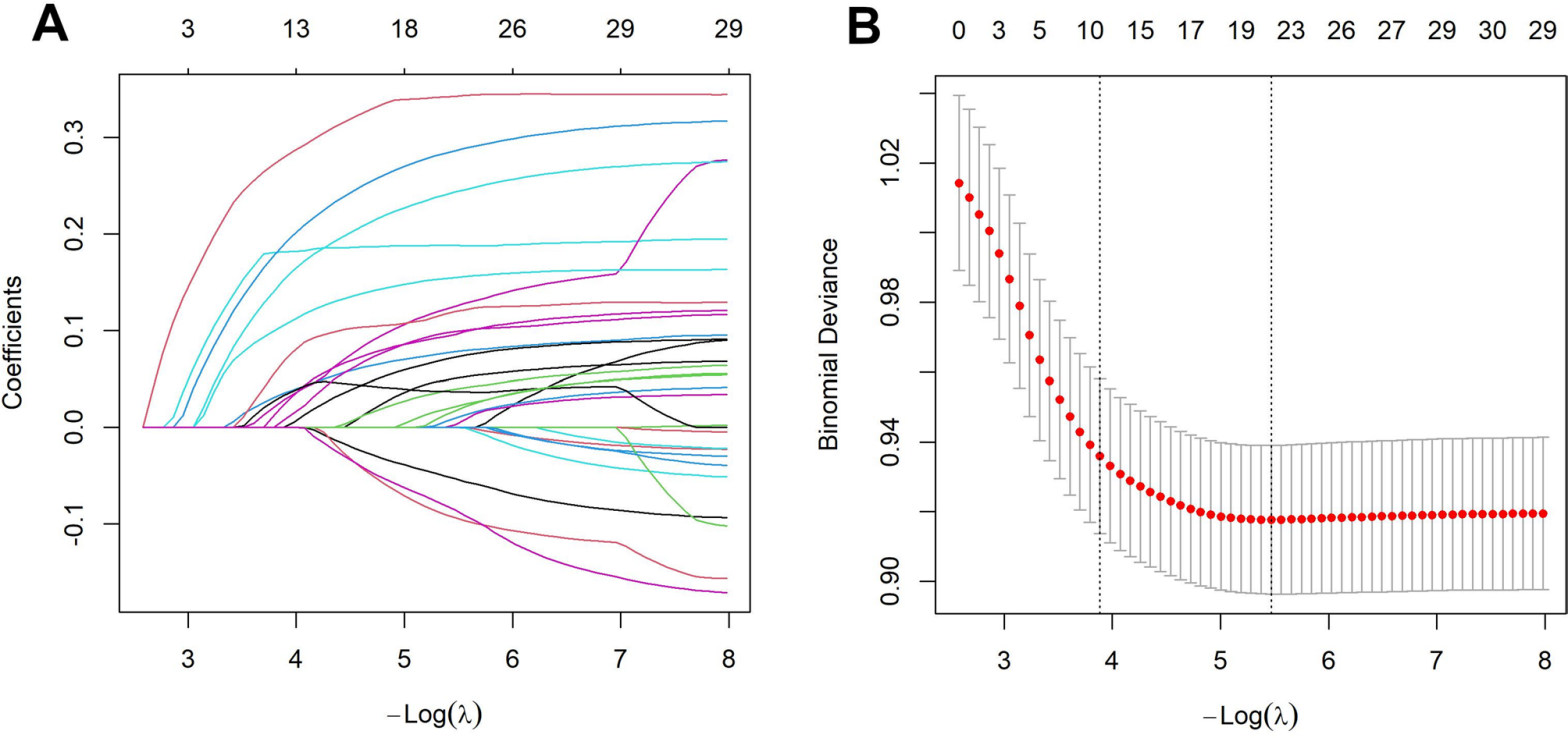
Variable selection from 31 potential predictors via LASSO regression. (A) Coefficient path diagram: illustrates how each variable’s regression coefficient progressively contracts towards zero as the penalty term (log(λ)) increases. The convergence process visually demonstrates LASSO’s variable selection capability. (B) Cross-validation error plot: Evaluates model performance under varying λ values via 10-fold cross-validation. The vertical dashed line indicates the optimal λ value selected using the ‘1 standard error’ criterion (lambda.1se), determining the final model’s variable selection. This process identified 11 predictors with non-zero coefficients.

To identify the strongest predictors of CMM, the training dataset was standardized. Using CMM as the dependent variable, LASSO regression assessed 31 potential predictors. The parameter λ was selected using the ‘1 standard error’ criterion (lambda.1se) to impose a stricter penalty constraint. This process retained 11 predictors with non-zero coefficients (Fig. 2): systolic blood pressure, BMI, waist circumference, fasting blood glucose, glycated haemoglobin, total cholesterol, triglycerides, uric acid, age, comorbidities, and pain.

**Fig. 3:**
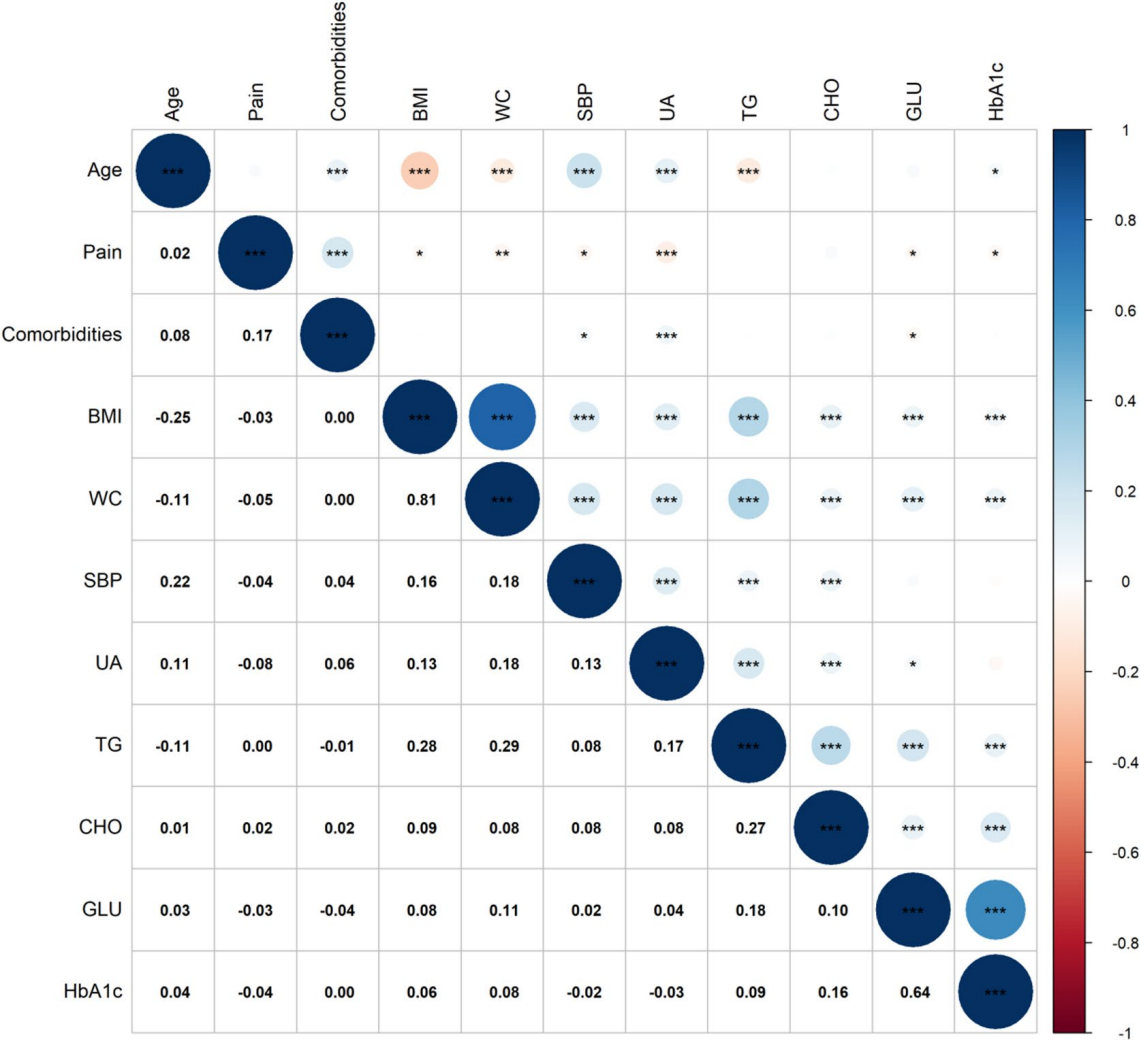
Correlation matrix of predictive variables. *: *p* < 0.05; **: *p* < 0.01; ***: *p* < 0.001. BMI: Body Mass Index; SBP: Systolic Blood Pressure; WC: Waist Circumference; TG: Triglycerides; CHO: Total Cholesterol; GLU: Glucose; UA: Uric Acid; HbA1c: Glycated Haemoglobin. Positive correlations are indicated in blue tones, negative correlations in red tones, with colour intensity reflecting correlation strength. Circle size is proportional to the absolute value of the correlation coefficient.

Figure 3 depicts the correlation matrix among the LASSO-selected predictors. Analysis revealed a strong correlation between waist circumference and BMI (*R* = 0.81), and a moderate correlation between fasting blood glucose and glycated haemoglobin (*R* = 0.64). To ensure model stability, parsimony, and clinical utility while minimizing overfitting risk, waist circumference and glycated haemoglobin were excluded. The final set of nine predictive variables was: systolic blood pressure, BMI, fasting blood glucose, total cholesterol, triglycerides, uric acid, age, comorbidities, and pain. This decision aligns with the principle of simplicity in clinical predictive model development.

### Construction of the XGBoost risk prediction model

**Fig. 4:**
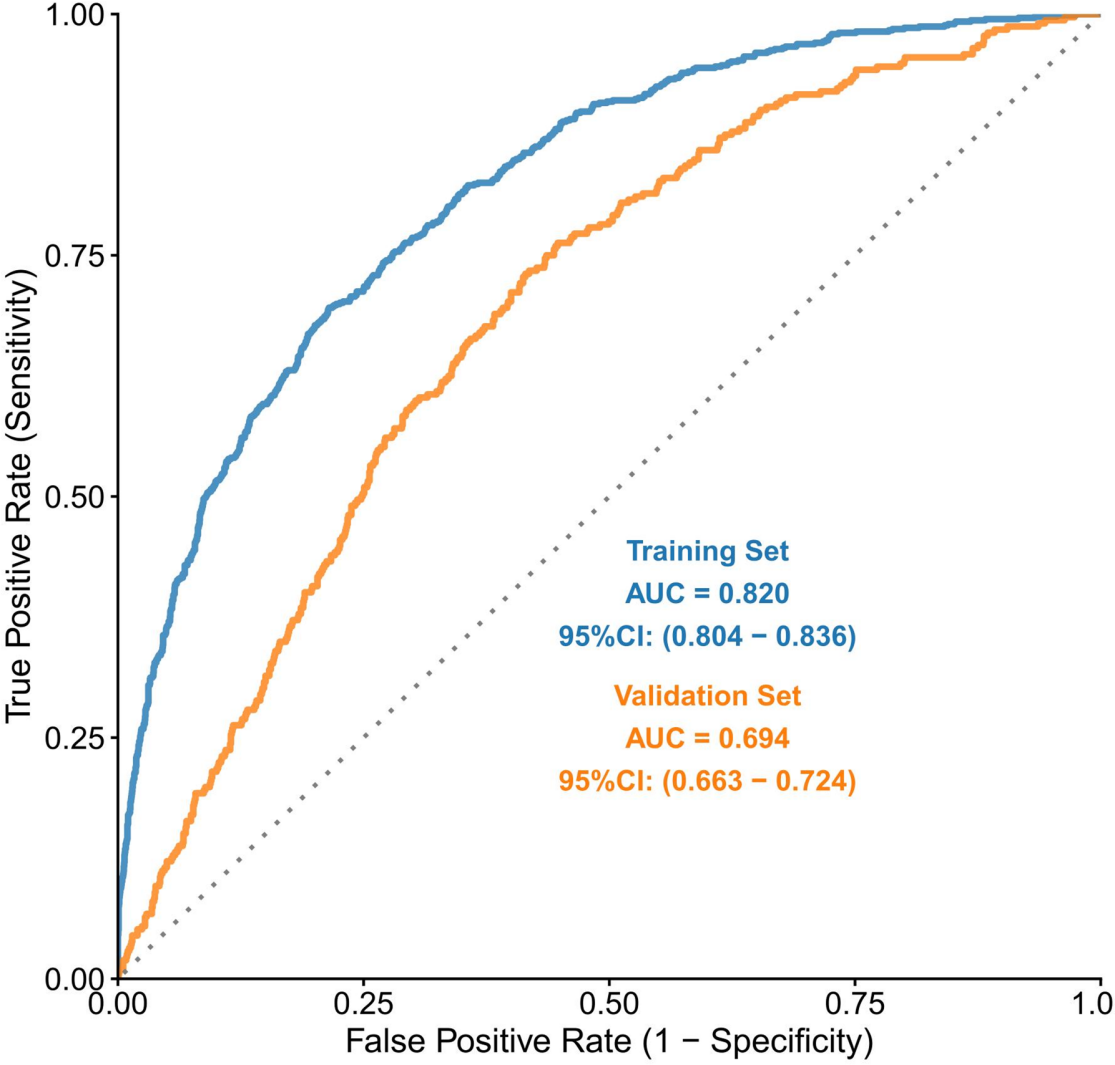
ROC curve of the XGBoost model.

A predictive model was constructed using the XGBoost algorithm with the nine finalized predictors. Bayesian optimization with 10-fold cross-validation yielded the optimal hyperparameter combination: nrounds = 199, subsample = 0.8, max depth = 2, eta = 0.144, and colsample bytree = 0.964. The final model, trained with these parameters, demonstrated excellent predictive capability on the training set (AUC = 0.820, 95% CI: 0.804–0.836) but moderate capability on the validation set (AUC = 0.694, 95% CI: 0.663–0.724), indicating overfitting (Fig. 4).

### SHapley additive exPlanations (SHAP) of the XGBoost model

**Fig. 5:**
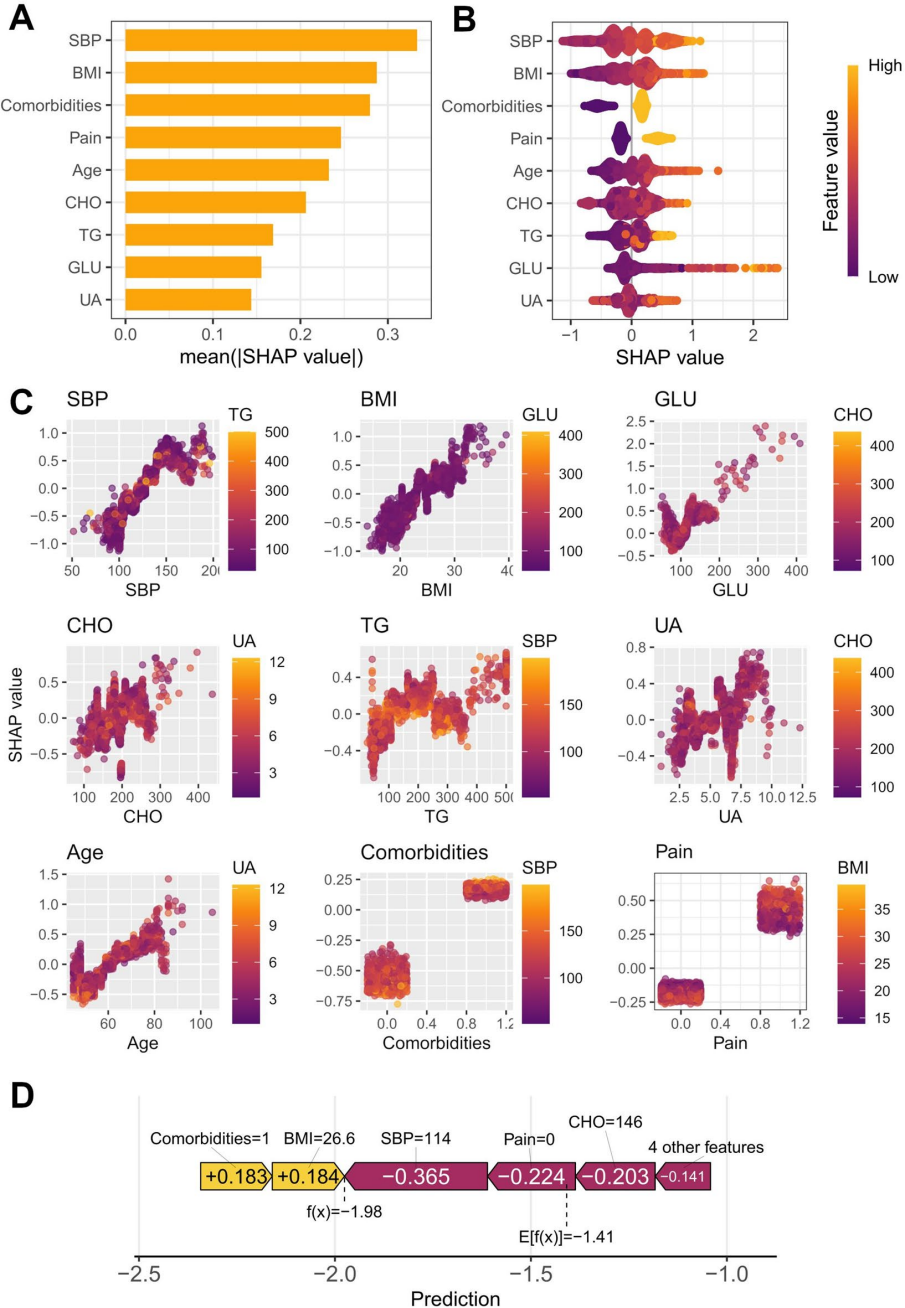
SHapley additive interpretability plots for CMM prediction. (**A**) SHAP Bar Plot. Variable importance in the predictive model displayed via SHAP values. (**B**) SHAP Summary Plot. SHAP values for model variables depicted as violin plots. Colour indicates feature value intensity, width denotes distribution density. (**C**) SHAP Dependence Plot. Each plot reveals the joint influence of two variables on model output, with colour intensity representing feature value magnitude. (**D**) SHAP Force Plot. Individual sample SHAP value analysis for CMM risk prediction, illustrating the marginal contribution of each predictor variable to the outcome.

We employed SHAP values to interpret the XGBoost model’s output. The SHAP Bar Plot (Fig. 5A) ranked predictors by their mean absolute SHAP values, identifying systolic blood pressure, BMI, and comorbidities as the most influential features. The SHAP Summary Plot (Fig. 5B) illustrated the direction and magnitude of each feature’s impact. SHAP Dependence Plots (Fig. 5C) and a SHAP Force Plot for an individual sample (Fig. 5D) provided detailed visualizations of feature effects at both population and individual levels, offering comprehensive insights into the model’s decision-making process.

### Construction of the logistic regression risk prediction model

**Fig. 6:**
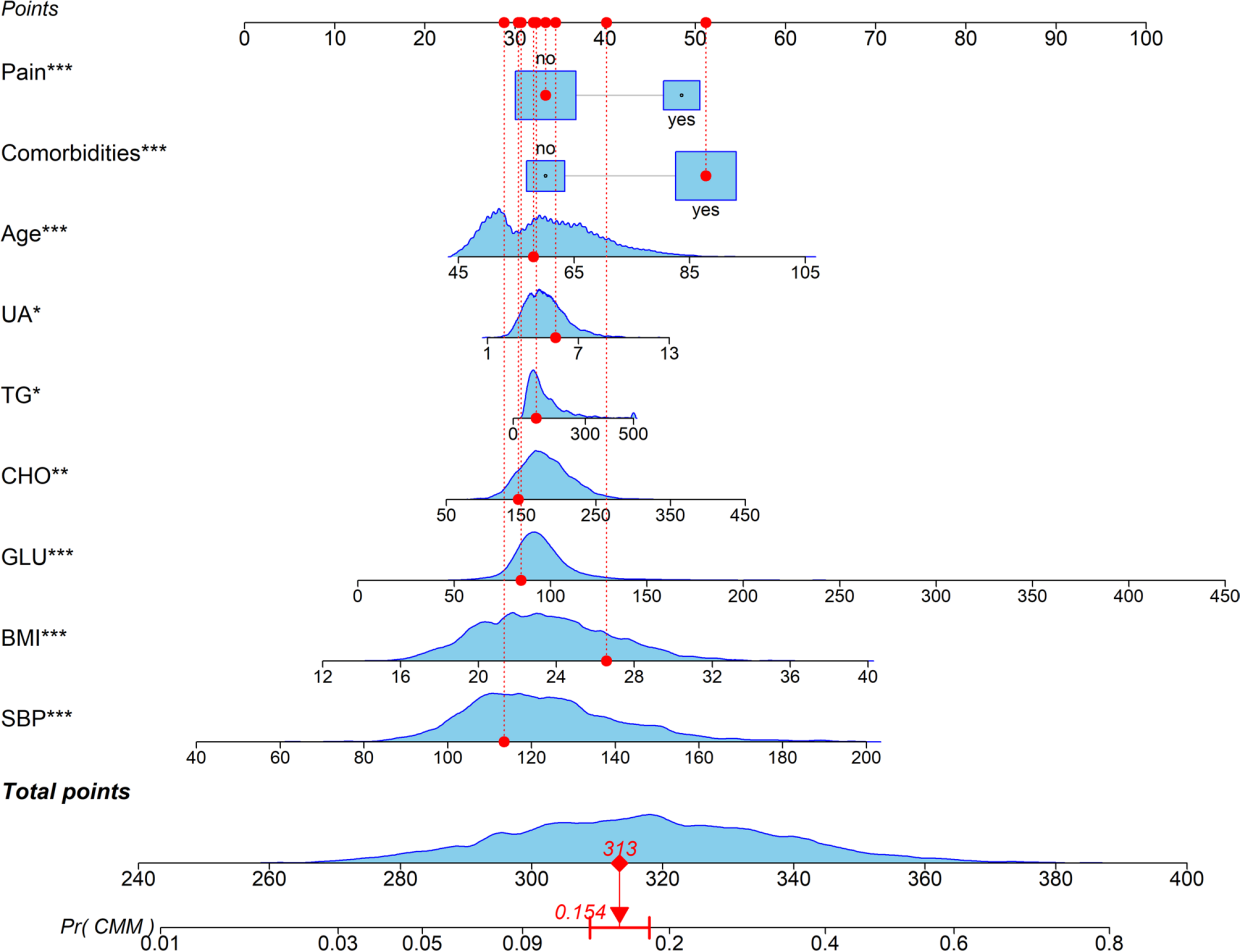
Nomogram for predicting CMM risk.

A predictive model was constructed using multivariable logistic regression analysis (Table 2) with the nine predictor variables. We developed a nomogram (Fig. 6) to visualize the model for predicting five-year CMM risk. To use the nomogram, the corresponding value for each variable is located, a vertical line is drawn to the points axis to obtain the score, the scores are summed to derive a total points value, and this total is projected onto the bottom scale to determine the individual’s CMM risk. For illustrative purposes, a total score of 313 points corresponded to an estimated 15.4% risk of developing CMM within five years.

**Table 2:** Multivariate Logistic Regression Analysis for Cardiometabolic Multimorbidity (CMM).

Variables	β	S.E	Z	*P*	OR (95% CI)
Intercept	−10.257	0.594	−17.266	<0.001	0.000 (0.000 ~ 0.000)
SBP	0.019	0.002	8.235	<0.001	1.019 (1.015 ~ 1.024)
BMI	0.089	0.013	6.633	<0.001	1.093 (1.065 ~ 1.123)
GLU	0.009	0.002	5.516	<0.001	1.009 (1.006 ~ 1.012)
CHO	0.003	0.001	2.768	0.006	1.003 (1.001 ~ 1.006)
TG	0.001	0.001	2.172	0.030	1.001 (1.001 ~ 1.002)
UA	0.069	0.032	2.174	0.030	1.072 (1.007 to 1.141)
Age	0.026	0.005	5.029	<0.001	1.027 (1.016 ~ 1.037)
Comorbidities					
No					1.000 (Reference)
Yes	0.734	0.111	6.636	<0.001	2.083 (1.677 ~ 2.587)
Pain					
No					1.000 (Reference)
Yes	0.623	0.093	6.714	<0.001	1.864 (1.554 ~ 2.236)

OR: Odds Ratio, CI: Confidence Interval.

### Comparison of predictive capabilities between the two models

**Fig. 7:**
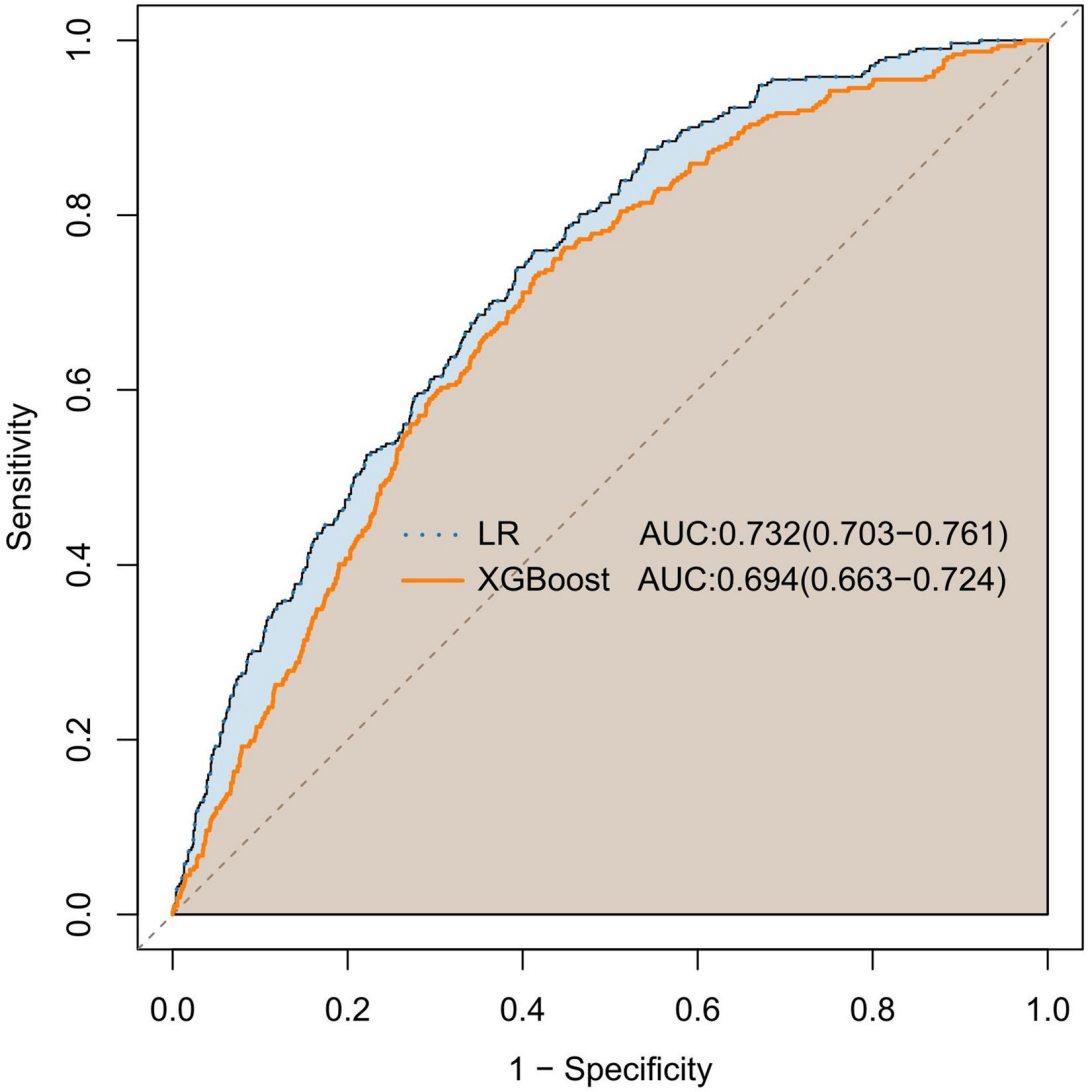
ROC curves comparing the predictive capabilities of the logistic regression and XGBoost models.

The predictive performance of the logistic regression (LR) and XGBoost models was compared on the validation set (*n* = 1,617). As shown in Fig. 7, the LR model achieved a higher AUC (0.732) compared to the XGBoost model (AUC = 0.694). Table 3 provides a detailed performance comparison. The LR model demonstrated superior sensitivity and a higher F1 score, indicating better overall performance and discriminative capability, particularly given the class imbalance in the data. These results established the logistic regression model as more effective for CMM risk assessment in this context.

**Table 3:** Performance comparison between XGBoost and logistic regression models.

Model	AUC	Accuracy	Sensitivity	Specificity	Precision	F1 score
XGBoost	0.694	0.792	0.067	0.966	0.318	0.111
LR	0.732	0.620	0.760	0.587	0.305	0.436

### Evaluation of the logistic regression risk prediction model

**Fig. 8:**
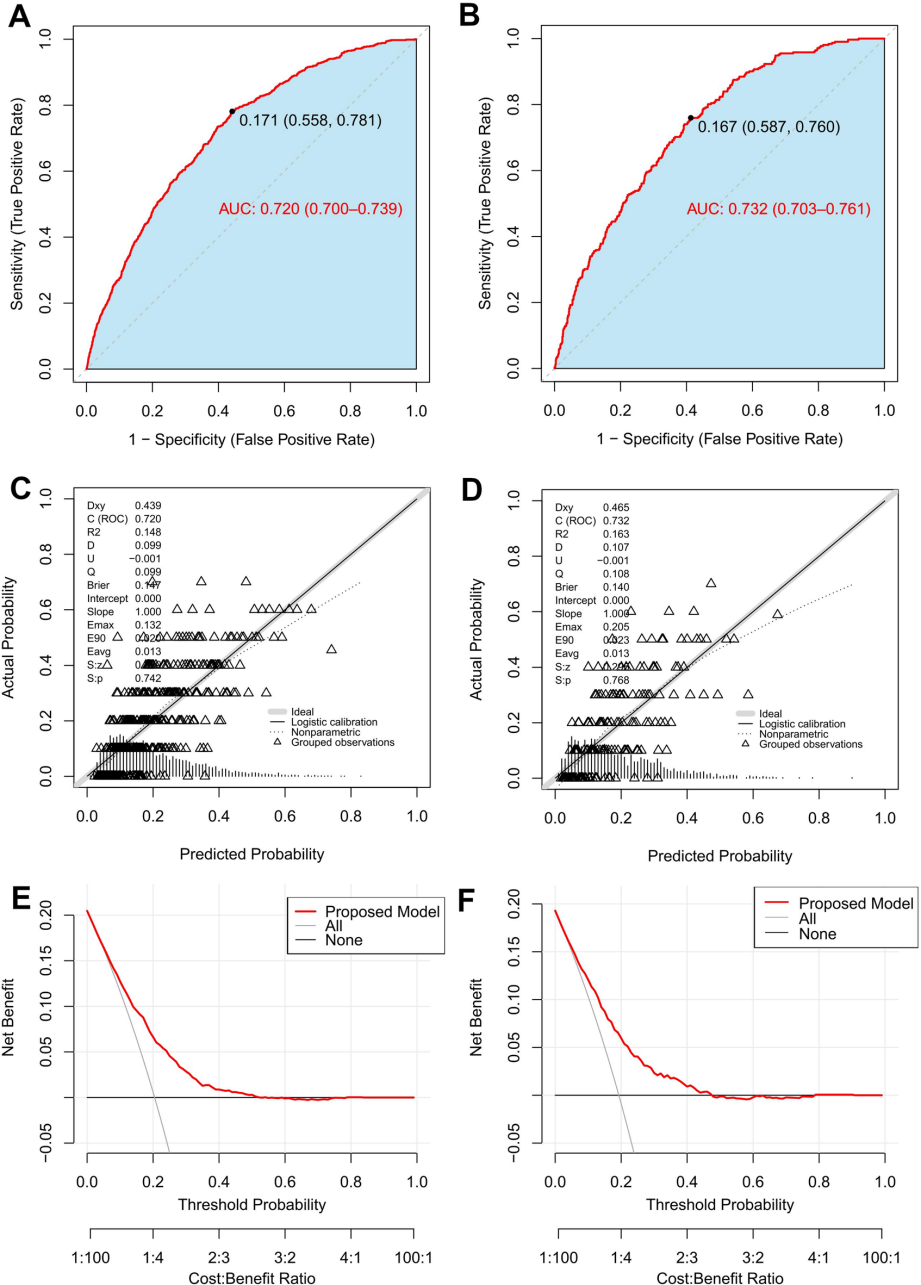
Evaluation of the logistic regression model. (**A**) ROC curve (training set). (**B**) ROC curve (validation set). (**C**) Calibration curve (training set). (**D**) Calibration curve (validation set). (**E**) Decision curve analysis (training set). (**F**) Decision curve analysis (validation set).

ROC curves for the training and validation sets (Fig. 8A and B) demonstrated the logistic regression model’s favourable and generalizable predictive capability. The AUC was 0.720 (95% CI: 0.700–0.739) for the training set and 0.732 (95% CI: 0.703–0.761) for the validation set. The optimal risk cut-off value was identified as 0.171, defining high CMM risk as a predicted probability exceeding 17.1%. A DeLong test indicated a statistically significant difference in AUC before and after excluding waist circumference and glycated haemoglobin in the validation set (0.741, 95% CI: 0.712–0.770 vs. 0.732, 95% CI: 0.703–0.761; *P* = 0.021). However, the absolute difference of 0.009 (95% CI: 0.001–0.016) was deemed clinically negligible, supporting the decision for a more parsimonious model.

To further determine whether our model significantly outperforms a simpler baseline risk score, we selected four clinically common predictors (systolic blood pressure, BMI, fasting glucose, and age) and constructed a corresponding model. The ROC curve for CMM prediction derived from this simplified model on the validation set is presented in Supplementary Material S2, yielding an AUC of 0.686 (95% CI: 0.653–0.718). A DeLong test confirmed that the full 9‑variable logistic regression model demonstrated superior predictive capability compared with this simpler model (AUC: 0.732 vs. 0.686; *P* < 0.001).

Calibration curves for both the training and validation sets (Fig. 8C and D) showed good agreement between predicted probabilities and observed outcomes, with a mean absolute error (MAE) of 0.013 (Brier score = 0.147) for the training set and an MAE of 0.014 (Brier score = 0.140) for the validation set.

Decision curve analysis (DCA) demonstrated the clinical utility of the logistic regression model across a range of risk thresholds. In both the training (Fig. 8E) and validation (Fig. 8F) sets, the red line representing the net benefit of using the model for decision-making was superior to the “treat all” or “treat none” strategies across a wide range of threshold probabilities, confirming its potential value in clinical practice.

### Subgroup analysis

To evaluate model applicability across age groups, we stratified the validation set into participants aged ≤ 60 years (*n* = 884) and > 60 years (*n* = 733). The model demonstrated good discriminative ability in both groups but performed better in the younger subgroup, with an AUC of 0.765 (95% CI: 0.728–0.802), compared to 0.705 (95% CI: 0.660–0.750) in the older subgroup. A DeLong’s test indicated that this difference was statistically significant (Z = 1.999, *p* = 0.046; see Supplementary Material S3).

To further assess model performance across different baseline cardiometabolic disease (CMD) statuses, we stratified the validation set into participants with no CMD at baseline (0 CMD, *n* = 777) and those with exactly one CMD at baseline (1 CMD, *n* = 824). In the 0 CMD subgroup, the model achieved an AUC of 0.737 (95% CI: 0.676–0.799), while in the 1 CMD subgroup, the AUC was 0.699 (95% CI: 0.662–0.737). A DeLong’s test showed no statistically significant difference between the two AUCs (*p* = 0.301) (see Supplementary Material S4).

### Sensitivity analysis

To assess potential reverse causation, a sensitivity analysis was performed by excluding individuals who developed CMM in 2018. The refitted model in the remaining validation cohort achieved an AUC of 0.776 (95% CI: 0.681–0.871). DeLong’s test showed no statistically significant difference from the primary model (AUC = 0.732; *p* = 0.437) (see Supplementary Material S5).

## Discussion

This study leveraged baseline data from the 2015 CHARLS to construct an effective prediction model for estimating the five-year risk of CMM onset. The final model incorporated nine key predictors strongly associated with CMM development in Chinese middle-aged and older adults: systolic blood pressure, BMI, fasting blood glucose, total cholesterol, triglycerides, uric acid, age, comorbidities, and pain. The use of longitudinal CHARLS data provides a robust, data-driven understanding of age-related CMM trends within the specific context of the Chinese population. The observed overall CMM incidence of 20.12% over five years highlights the critical public health importance of early identification and intervention. The inclusion of physical condition indicators like comorbidities and pain as predictive variables represents a novel aspect of this research.

The five‑year CMM incidence of 20.12% in our cohort aligns with the epidemiological pattern of cardiometabolic disease burden in the aging Chinese population^29^. For instance, a large‑scale prospective cohort study with 11.2 years of follow‑up reported that among individuals free of CMD at baseline, 19.02% developed at least one CMD, of whom 16.15% progressed to CMM^30^. In terms of predictive performance, previous models that relied primarily on single or composite indicators not only demonstrated limited predictive capacity (AUC < 0.70)^9,31^ but, more importantly, failed to establish an effective risk‑scoring tool to guide CMM risk stratification and individualized intervention. This further highlights the value of the CMM prediction model developed in the present study.

Our approach to algorithm and variable selection was guided by three principles: predictive capability, clinical utility, and handling of multidimensional data complexity. While XGBoost is renowned for its predictive power, the logistic regression model, visualized via a nomogram, offers superior interpretability and ease of clinical implementation. From an initial pool of 31 predictors, LASSO regression and subsequent correlation analysis efficiently refined the variable set. This process led to the exclusion of waist circumference and glycated haemoglobin due to their high correlations with BMI and fasting glucose, respectively, which enhanced model parsimony and stability. The statistically significant but clinically negligible impact of this exclusion on the AUC (as reported in the Results) justifies our pursuit of a simpler, more robust model for clinical application.

The nine predictors were incorporated into both the XGBoost and logistic regression models. The XGBoost model showed signs of overfitting, with excellent performance on the training set but markedly lower performance on the validation set (training AUC = 0.820 vs. validation AUC = 0.694), which would significantly compromise its generalizability beyond the CHARLS cohort. Given the suboptimal validation performance of the XGBoost model, the subsequent SHAP analysis should be interpreted with caution. Its primary value lies in offering an exploratory illustration of feature interactions within a complex, overfitted model, providing preliminary insights into which variables were prioritized during the fitting process. We explored several potential causes for this discrepancy^32–35^. First, despite the use of Bayesian optimization with 10‑fold cross‑validation, the effective sample size relative to the model’s complexity may have been insufficient. XGBoost, with its capacity to model complex non‑linear relationships and interactions through multiple trees and depths, inherently possesses a high ability to learn noise in the training data. Given the limited sample size in the training set, the data may have been insufficient to robustly support the complex patterns the algorithm attempted to capture, resulting in strong in‑sample performance but poor generalization. Second, the clinical relationships in our dataset for predicting CMM risk at the population level may be predominantly linear or additive. Consequently, XGBoost’s strength in capturing intricate interactions and non‑linearities may have fitted spurious, sample‑specific patterns that did not hold in the validation set. Furthermore, given the moderate sample size, the linear decision mechanism of logistic regression proved to be a more stable and appropriate choice for this particular prediction task. Although cross‑validation is a robust technique for hyperparameter tuning, it is not infallible. It primarily guards against overfitting to a single train‑test split, but performance can still be overestimated if the overall sample size is limited or if unaccounted heterogeneity exists (as indicated by some differences between the cohorts in the baseline table). In contrast, the logistic regression model demonstrated robust and consistent discriminative ability across both training and validation sets, along with good calibration and clinical utility. Visualization of this model via a nomogram effectively bridges statistical prediction with routine clinical practice. Furthermore, in a sensitivity analysis that excluded individuals who developed CMM in 2018, the model exhibited predictive performance consistent with the primary findings, effectively mitigating concerns regarding potential reverse causation. Based on its stable predictive performance and clinical interpretability—and despite the theoretical potential of XGBoost to achieve higher predictive ceilings or of further reducing overfitting by using shallower trees or stronger regularization—the logistic regression model was selected as the final predictive tool for this study.

The nine predictors in our model comprehensively reflect core pathophysiological pathways underlying CMM, encompassing metabolic dysregulation, aging, multimorbidity burden, and symptom load.

Elevated systolic blood pressure (SBP) was a leading predictor, consistent with its established role as a primary driver of atherosclerosis and cardiovascular events. Research indicates that each 20 mmHg increase in SBP is associated with a more than twofold risk of stroke mortality^36^, while antihypertensive treatment significantly reduces cardiovascular risk^37^. Hypertension promotes endothelial dysfunction and target organ damage, forming a critical link between cardiovascular and metabolic diseases^38^. In China’s aging population, hypertension often clusters with glucose and lipid metabolism disorders, synergistically increasing the risk of heart disease and stroke. The low awareness and control rates of hypertension in China underscore the urgent need for improved blood pressure management to mitigate CMM risk.

BMI served as a key indicator of adiposity. Excess body fat, particularly visceral fat, elevates CMM risk by promoting insulin resistance and chronic low-grade inflammation^39,40^. Adipose tissue functions as an active endocrine organ, secreting inflammatory cytokines and adipokines that directly contribute to insulin resistance, hypertension, and dyslipidaemia^41^. Insulin resistance itself is considered a central mechanism in cardiometabolic disorders^42^.

Abnormalities in fasting blood glucose, total cholesterol, triglycerides, and uric acid are hallmark indicators of metabolic disease. Dysregulated glucose and lipid metabolism significantly elevates CMM risk, correlating with insulin resistance and inflammation^43,44^. Hyperglycaemia causes direct vascular damage via advanced glycation end-products and oxidative stress^45^. Elevated total cholesterol (especially LDL-C) drives atherosclerotic plaque formation, while high triglycerides are closely linked to insulin resistance and contribute to residual cardiovascular risk^46^. Uric acid emerged as an independent predictor, reflecting its growing recognition beyond gout; hyperuricaemia is bidirectionally linked to hypertension, insulin resistance, and kidney disease^47^, potentially promoting CMM through inflammatory pathways and endothelial dysfunction^48^.

This study identified pain as a significant predictor of CMM risk. It is important to note, however, that the pain variable included was non-specific, broadly defined as the presence of pain in any of 14 anatomical locations without further stratification by severity, duration, or chronicity. This constitutes a major limitation of the current analysis. The heterogeneity inherent in this measure likely contributed to its emergence as a predictor while simultaneously constraining detailed clinical interpretation. Nevertheless, its retained significance after rigorous variable selection suggests a meaningful, albeit non-specific, association with CMM risk. Several potential explanations are proposed. First, chronic pain—irrespective of its location—may act as a persistent stressor, leading to dysregulation of the hypothalamic-pituitary-adrenal (HPA) axis and increased sympathetic tone. This state can elevate cortisol levels, promote systemic inflammation, and worsen metabolic control (e.g., insulin resistance), thereby creating a shared biological pathway toward CMM^49,50^. Second, pain often leads to reduced physical activity and functional decline, which are independent risk factors for weight gain, poor glycemic control, and cardiovascular deconditioning, thus exacerbating cardiometabolic risk profiles^51^. Furthermore, the experience of pain may reflect broader issues of allostatic load or frailty, wherein accumulated physiological dysregulation across multiple systems manifests as both somatic complaints (e.g., pain) and susceptibility to chronic diseases such as CMM^52^. Therefore, within our predictive model, pain may serve less as a direct causal agent and more as a clinically accessible proxy for a systemic state of physiological dysregulation, chronic stress, or compromised health that predisposes individuals to CMM. Future studies should aim to collect more precise pain metrics (e.g., severity, duration using standardized tools like the Visual Analog Scale or pain diaries) to disentangle these relationships and determine whether specific pain phenotypes confer higher CMM risk.

Age is an irreversible risk factor for most chronic diseases, reflecting cumulative exposure and physiological decline^53^. Our findings reaffirm the strong association between advancing age and cardiovascular-metabolic abnormalities^54^. The impact of age on CVD risk is also modulated by sex, with risks increasing markedly in women after menopause^55^. Older age also brings heightened risks of comorbidities and diminished functional capacity, which collectively influence metabolic health and cardiovascular disease development. Of note, our age-stratified analysis revealed that the model exhibited stronger discriminative ability in individuals aged ≤ 60 years (AUC = 0.765) than in those > 60 years (AUC = 0.705). This difference may be explained by the generally higher burden of age-related risk factors and subclinical disease among older adults (>60 years), which elevates risk scores across the population and makes it more difficult to distinguish those who will progress to CMM within five years^56^. In addition, the pathophysiology of incident CMM in older adults may involve a greater contribution from geriatric-specific factors (e.g., frailty) that are not fully captured by our primarily metabolism- and symptom-based predictors^57,58^. Consequently, although the model provides clinically useful risk stratification across the entire age range studied, it appears particularly suitable for identifying high-risk individuals in midlife.

Furthermore, our subgroup analysis based on baseline cardiometabolic disease status demonstrated that the model maintained moderate and statistically comparable predictive performance both in individuals with no CMD and in those with a single existing CMD. This indicates that the model not only identifies individuals at elevated risk due to pre-existing conditions but also enables genuine risk stratification among seemingly healthy adults prior to any CMD onset.

The presence of existing comorbidities signifies a higher allostatic load and prolonged exposure to shared risk factors (e.g., aging, genetics, adverse lifestyle, chronic inflammation). This results in diminished physiological reserve and increased vulnerability, substantially elevating CMM risk^52^. Comorbidities can indirectly promote poor control of glucose, blood pressure, and lipids by limiting functional capacity, affecting drug metabolism, and exacerbating systemic inflammation^59^. Thus, incorporating comorbidities captures signals of overall health deterioration beyond traditional metabolic indicators, providing a more holistic risk assessment that aligns with clinical experience.

The primary strength of our prediction model is its shift from reactive management of diagnosed conditions toward proactive risk stratification for multimorbidity. In current practice, clinicians typically address elevated blood pressure, glucose, or lipid levels when detected. However, individuals with only modest elevations across several risk factors may not receive intensive intervention, even though they carry a substantial cumulative risk for cardiometabolic multimorbidity (CMM). Visualized through a nomogram, our model quantifies this synergistic risk and demonstrates clinical benefit across various risk thresholds. This tool helps clinicians identify high‑risk individuals who might otherwise be overlooked and facilitates timely lifestyle or pharmacological interventions to prevent the progression from isolated risk factors to overt multimorbidity.

This study has several limitations. First, although the predictors were selected using rigorous methodological criteria, the model demonstrated only moderate discriminative ability (AUC = 0.732), partly due to the inability to incorporate variables with high missing rates such as detailed physical activity and mental health status. However, the primary objective of this research was to develop a tool for population-level screening and early risk identification rather than for disease diagnosis. According to established research practice in public health and preventive medicine, an AUC above 0.70 is generally considered acceptable for preliminary risk triage and prioritization of interventions^60–63^. Second, although the model performed well in internal validation, external validation could not be conducted due to the lack of a suitable independent dataset. Future studies should incorporate data from community hospitals or other cohorts for external validation to further substantiate the model’s generalizability and clinical applicability.

## Conclusion

This study integrated multidimensional features to predict CMM risk, ultimately constructing and validating a logistic regression model based on nine readily available predictive variables. Visualized through an intuitive nomogram, this tool accounts for the complex pathophysiology of CMM and overcomes the limitations of prior studies reliant on single or composite indicators. It enhances the precision of individualized risk assessment and addresses the clinical need for efficient identification of high-risk populations. Consequently, our model provides a reliable, practical, and individualized CMM risk prediction tool for middle-aged and older adults, healthcare professionals, and clinical guideline developers in China. It offers evidence-based support for the early intervention and management of high-risk populations, with the ultimate goal of reducing the substantial health and economic burden of CMM in China’s aging society.

## Supplementary Information

Below is the link to the electronic supplementary material.

Supplementary Material 1

Supplementary Material 2

Supplementary Material 3

Supplementary Material 4

Supplementary Material 5

Supplementary Material 6

## Data Availability

The data supporting this study are available from the official CHARLS website: http://charls.pku.edu.cn/. This survey data is openly available to the public. The datasets generated and/or analyzed during the current study are available from the corresponding author upon reasonable request.
