# Supplementary material for: Development and validation of a cardiometabolic multimorbidity prediction model in middle-aged and older adults
Source: Sci Rep. 2026 Mar 12;16:13300. doi: 10.1038/s41598-026-44213-0 (PMC13106848; doi:10.1038/s41598-026-44213-0)
Supplement: Supplementary file 1 — Supplementary Material 1 [file 41598_2026_44213_MOESM1_ESM.docx]

## S1. Disease Combinations in CMM

| Combination_Type | Disease_Combination | Count | Percentage |
| --- | --- | --- | --- |
| 2 -way combination | Diabetes + Dyslipidaemia | 64 | 1.188 |
| 2 -way combination | Diabetes + Heart Disease | 30 | 0.557 |
| 2 -way combination | Diabetes + Stroke | 9 | 0.167 |
| 2 -way combination | Dyslipidaemia + Heart Disease | 83 | 1.540 |
| 2 -way combination | Dyslipidaemia + Stroke | 22 | 0.408 |
| 2 -way combination | Heart Disease + Stroke | 18 | 0.334 |
| 2 -way combination | Hypertension + Diabetes | 80 | 1.485 |
| 2 -way combination | Hypertension + Dyslipidaemia | 221 | 4.101 |
| 2 -way combination | Hypertension + Heart Disease | 160 | 2.970 |
| 2 -way combination | Hypertension + Stroke | 68 | 1.262 |
| 3 -way combination | Diabetes + Dyslipidaemia + Heart Disease | 9 | 0.167 |
| 3 -way combination | Diabetes + Dyslipidaemia + Stroke | 7 | 0.130 |
| 3 -way combination | Diabetes + Heart Disease + Stroke | 0 | 0.000 |
| 3 -way combination | Dyslipidaemia + Heart Disease + Stroke | 6 | 0.111 |
| 3 -way combination | Hypertension + Diabetes + Dyslipidaemia | 62 | 1.151 |
| 3 -way combination | Hypertension + Diabetes + Heart Disease | 25 | 0.464 |
| 3 -way combination | Hypertension + Diabetes + Stroke | 7 | 0.130 |
| 3 -way combination | Hypertension + Dyslipidaemia + Heart Disease | 93 | 1.726 |
| 3 -way combination | Hypertension + Dyslipidaemia + Stroke | 34 | 0.631 |
| 3 -way combination | Hypertension + Heart Disease + Stroke | 10 | 0.186 |
| 4 -way combination | Diabetes + Dyslipidaemia + Heart Disease + Stroke | 7 | 0.130 |
| 4 -way combination | Hypertension + Diabetes + Dyslipidaemia + Heart Disease | 25 | 0.464 |
| 4 -way combination | Hypertension + Diabetes + Dyslipidaemia + Stroke | 11 | 0.204 |
| 4 -way combination | Hypertension + Diabetes + Heart Disease + Stroke | 2 | 0.037 |
| 4 -way combination | Hypertension + Dyslipidaemia + Heart Disease + Stroke | 24 | 0.445 |
| 5 -way combination | Hypertension + Diabetes + Dyslipidaemia + Heart Disease + Stroke | 7 | 0.130 |
