# Supplementary figures and images for: Development and validation of a cardiometabolic multimorbidity prediction model in middle-aged and older adults
Source: Sci Rep. 2026 Mar 12;16:13300. doi: 10.1038/s41598-026-44213-0 (PMC13106848; doi:10.1038/s41598-026-44213-0)

## S2. ROC Curve of the Model with SBP, BMI, GLU, and Age on the Validation Set


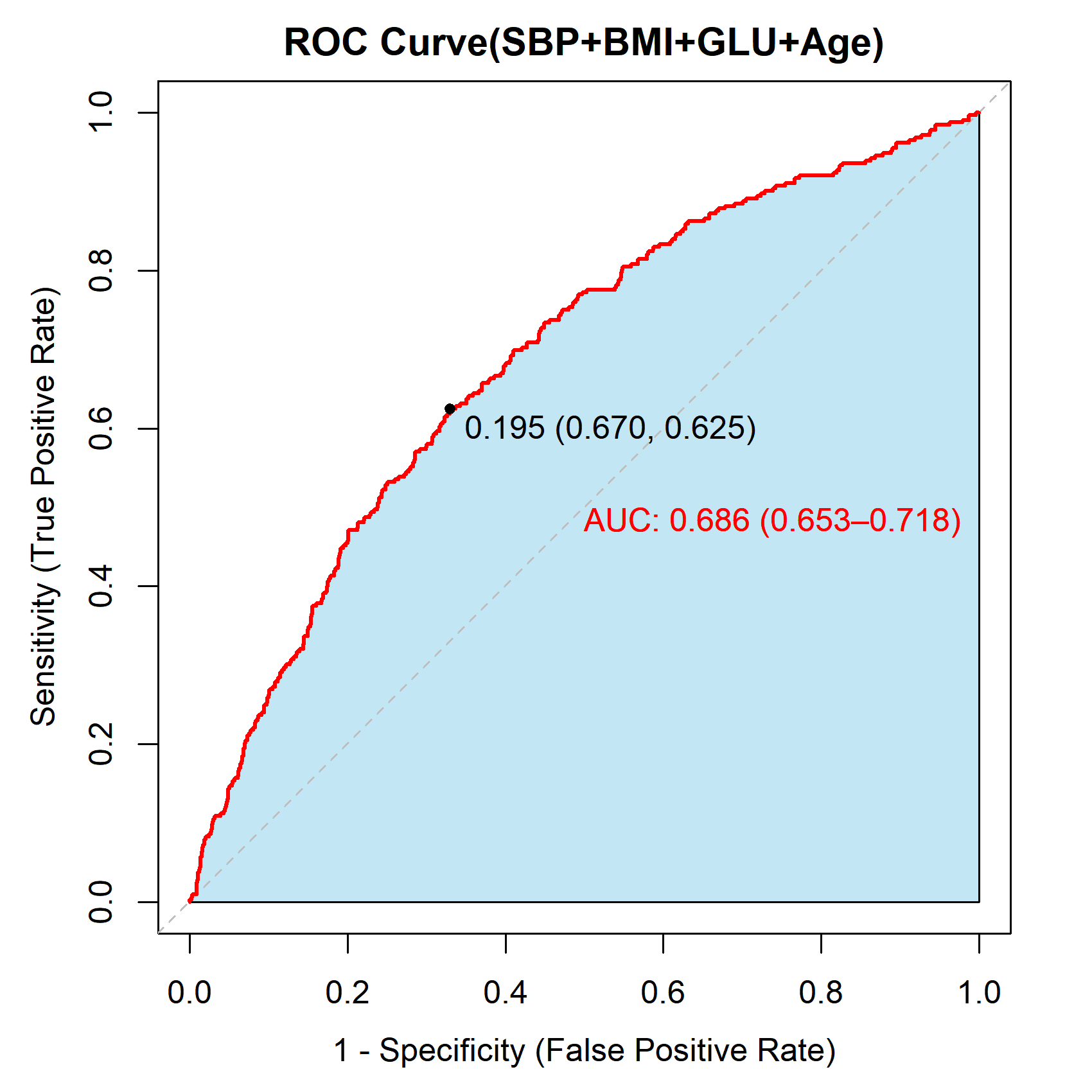

Supplement: Supplementary file 2 — Supplementary Material 2 [file 41598_2026_44213_MOESM2_ESM.docx]

## S5. ROC Curve of the Model After Excluding Individuals Who Developed CMM in 2018


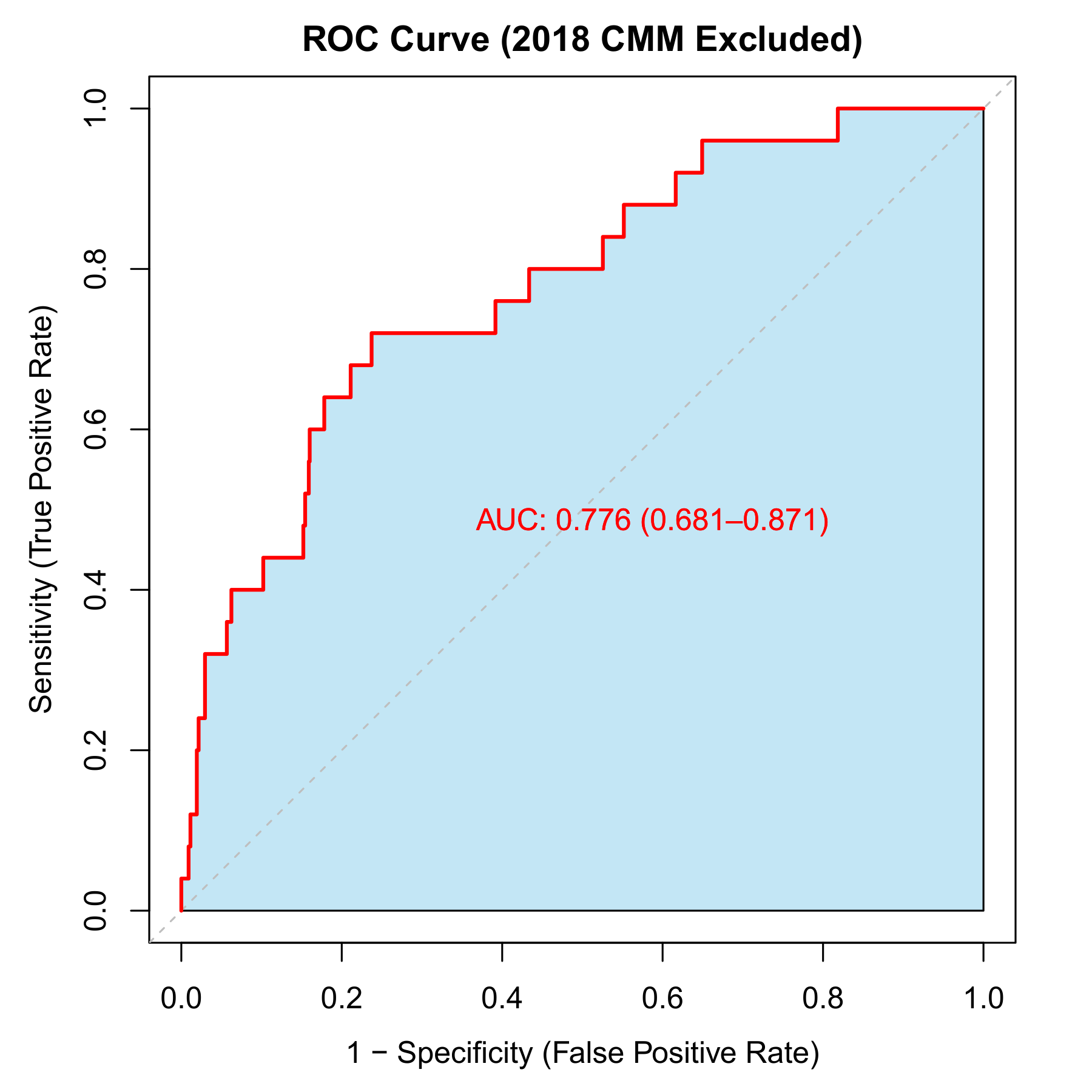

Supplement: Supplementary file 4 — Supplementary Material 4 [file 41598_2026_44213_MOESM4_ESM.docx]

## S3. ROC Curves of the Model Across Age Subgroups


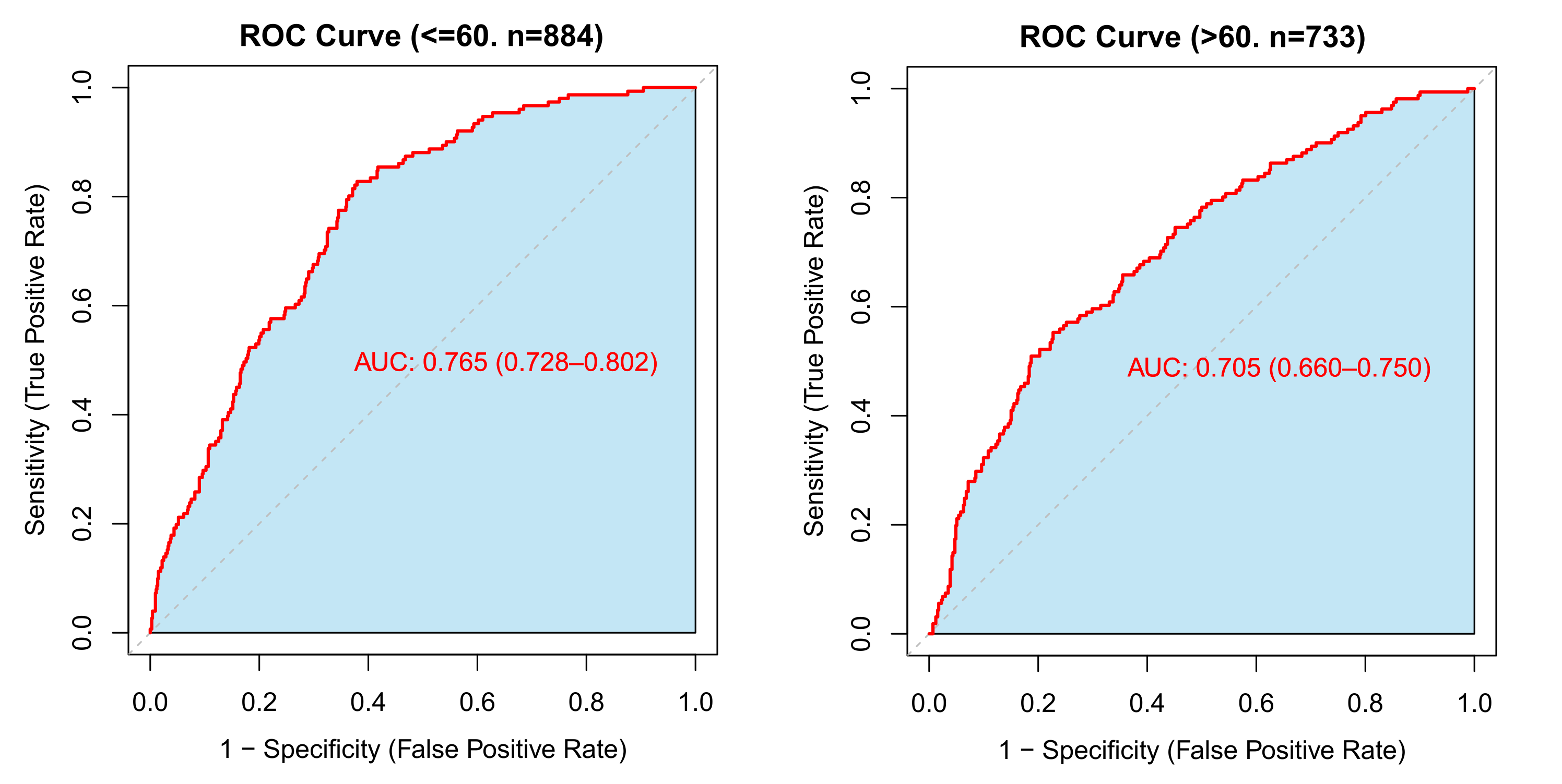

Supplement: Supplementary file 5 — Supplementary Material 5 [file 41598_2026_44213_MOESM5_ESM.docx]

## S4. ROC Curves of the Model in the 0 CMD and 1 CMD Subgroups


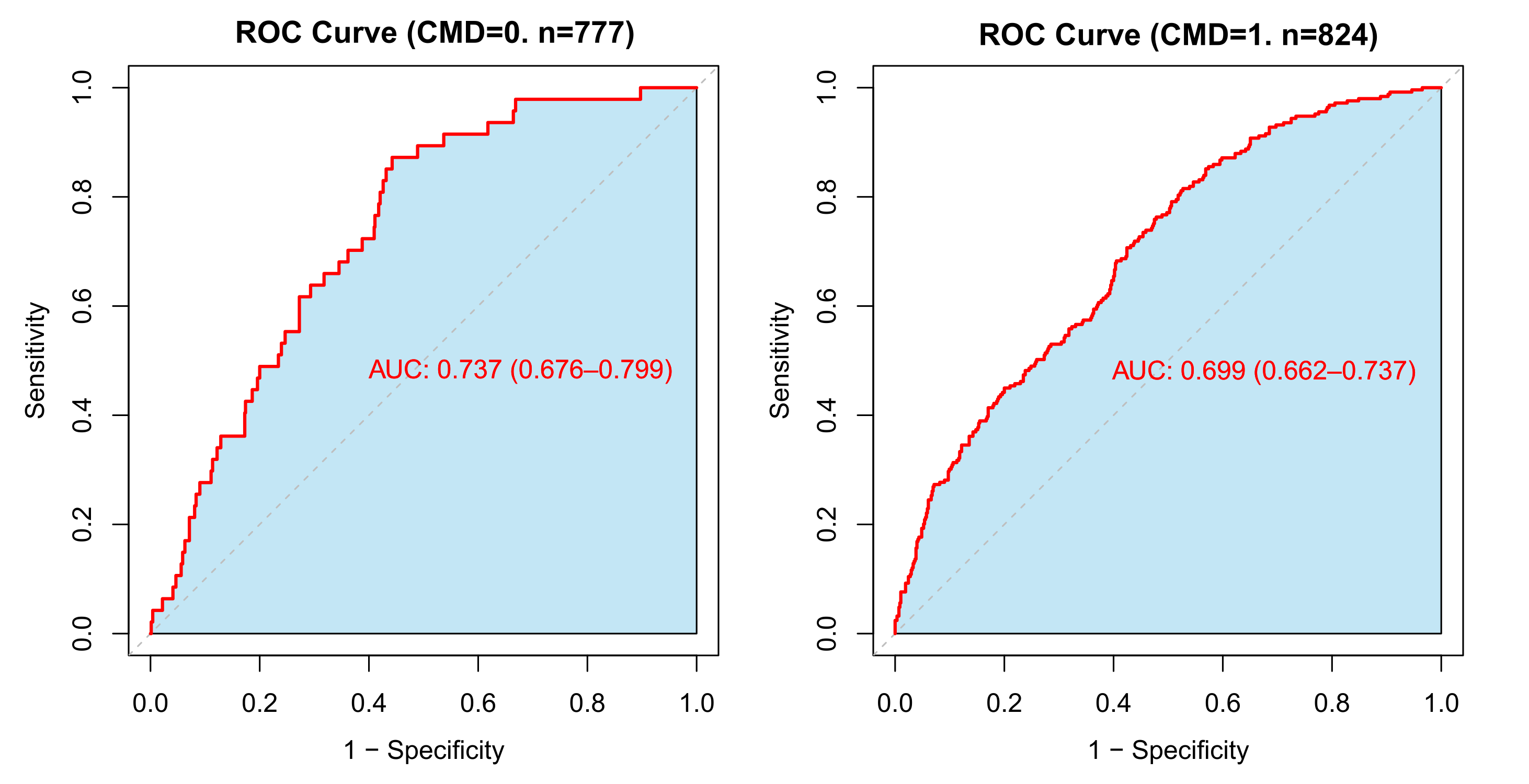

Supplement: Supplementary file 6 — Supplementary Material 6 [file 41598_2026_44213_MOESM6_ESM.docx]
